# Short-term health system responses to epidemics across hard-to-reach areas in sub-Saharan Africa: a scoping review

**DOI:** 10.1186/s40249-026-01463-4

**Published:** 2026-05-28

**Authors:** Annette A. Murunga, Ben Ngoye, Francis P. Wafula

**Affiliations:** Institute of Healthcare Management, Strathmore Business School, Nairobi, Kenya

**Keywords:** Epidemic preparedness, Infectious diseases, Health system resilience, Governance, Policy response, Sub-Saharan Africa

## Abstract

**Background:**

Epidemics and disease outbreaks continue to threaten public health security in sub-Saharan Africa (SSA), disproportionately impacting impoverished and hard-to-reach populations. Although many country-specific studies exist, few syntheses have examined short-term health system responses to epidemics in hard-to-reach areas of SSA and their effects on health equity and resilience. This scoping review consolidates regional evidence on structural and policy-relevant lessons for enhancing health system preparedness and epidemic management in resource-limited settings.

**Methods:**

A scoping review was conducted in accordance with the PRISMA-ScR guidelines. Four electronic databases (PubMed, Cochrane Library, CAB Direct, and Google scholar) and grey literature sources for studies published between 2012 and 2022. Eligible studies reported short-term (immediate or early-phase) health system responses to epidemic-prone infectious diseases in SSA. Data were extracted thematically using Excel and analysed using a modified Donabedian framework encompassing structures, processes, and outcomes.

**Results:**

Fifteen studies met the inclusion criteria and examined responses to Coronavirus 2019 (COVID-19), Ebola Virus Disease (EVD), and other epidemic-prone infections. Common weaknesses identified included shortages of trained healthcare workers, limited financial resources, poor leadership and coordination, and weak information systems. However, countries such as Rwanda, Ethiopia, Nigeria, and Uganda demonstrated adaptive governance, decentralised coordination, and the use of digital tools to improve surveillance, communication, and service delivery. Strong community engagement helped reduce stigma and increased adherence to control measures, especially in rural and underserved areas. Countries that incorporated epidemic response into existing primary healthcare and routine services achieved better equity and system resilience.

**Conclusion:**

The scoping review underscores strong evidence for incorporating epidemic preparedness into the broader health system. Policy focus areas include enhancing leadership and governance, establishing swift response mechanisms at subnational levels, and utilising technology for real-time data and coordination. Regional collaborations like those facilitated by the Africa CDC can improve collective resilience. Going forward, policies should prioritise not just emergency response but also ongoing investment in flexible, learning health systems that can withstand shocks while consistently providing essential services.

**Supplementary Information:**

The online version contains supplementary material available at 10.1186/s40249-026-01463-4.

## Background

The Coronavirus (Covid-19) pandemic pushed the world to the brink of a social and economic meltdown, triggering questions around how well the global health community is prepared to handle major epidemics. The world had to deal with a virus that created chaos across every aspect of life [1]. That said, we cannot claim full ignorance on the risk of such calamities. Health system inequities and poor resourcing of healthcare, especially across low- and middle-income settings, put their populations at risk. One type of population group that is particularly ignored and underfunded, yet carries a substantial risk of pandemics, is those living in hard-to-reach areas across Africa. We define hard-to-reach areas as places where it would be hardest or take longest for someone to access basic humanitarian services (such as health clinics and hospitals), lack of functioning transport links and infrastructure, as well as terrain difficulty [2].

Health systems in LMICs are barely able to handle those in high-access areas, such as urban and more affluent areas. Major gaps also exist in key areas like surveillance, emergency preparedness and response, risk communication and effective control and management of points of entry [3]. A World Health Organisation (WHO) evaluation reported that most SSA countries are not equipped to respond to sudden shocks to health systems, yet the region often suffers isolated epidemics [4]. Worse still, broader dynamics continue to contribute to the emergence and re-emergence of outbreaks of new diseases and antimicrobial resistance [5]. Despite outbreaks like Severe Acute Respiratory Syndrome (SARS), Cholera, Chikungunya, Dengue Fever, Rift Valley Fever and Ebola providing warning signs and testing countries’ ability to respond [6], Covid-19 still found most low- and middle-income countries (LMICs) unprepared. Poor emergency response strategies were made worse by inadequate laboratory and epidemiological surveillance systems and economic, social, and political disincentives [7].

While COVID-19 presented a particularly unique challenge for most countries, we cannot fully appreciate the path-dependent challenges affecting SSA without going back in time to understand how countries have responded to other types of outbreaks. We are particularly keen to document the immediate or short-term response to better understand policy agility and capacity to learn, adapt and change when the need does not appear to be urgent. We know that because most SSA countries focus on urgent, rather than important matters, their ability to handle COVID-19, which is both an urgent and important matter, was compromised. We argue that by understanding immediate policy responses to outbreaks in low-priority areas, we may be able to characterise and address the pain points that prevent countries from prioritising and establishing mechanisms to nip outbreaks in the bud before major calamity. We argue that while effort has gone towards examining health sector response to specific outbreaks, not enough attention has gone towards characterising commonalities in immediate response to disease of different kinds, and how the immediate actions translate into (or fail to translate into) medium- and longer-term strategies. Examining the causal relationships between the response activities and the broader health system change is crucial, if the global health community is to find a lasting solution to epidemics. This review characterises immediate responses to epidemics across Sub-Saharan Africa and examines how crucial components of the response morph (or fail to morph) into medium- and longer-term health system changes to avert recurrence or improve future response. Our scoping review also aimed to provide an overview of existing evidence on immediate health system responses to epidemics in SSA and answer the following questions: (1) What is the context of the health system (service delivery, Human Resources, Leadership and Governance, Financing, Medicines, and Information Tech) regarding pandemic preparedness and management in SSA? (2) What are the main functions and activities of the health system in SSA, and what diversity of approaches in terms of policies, protocols, communication, community participation, and surveillance were used to manage epidemics? (3) What were the outcomes of the various approaches and models in early detection of diseases, reduced disease impact on the population, change in health behaviour, and strengthened health system?

## Methods

The scoping review was conducted following the methodological framework by Arksey and O’Malley and adhered to the Preferred Reporting Items for Systematic Reviews and Meta-Analyses extension for Scoping Reviews (PRISMA-ScR) guidelines, as well as a modified Donabedian framework encompassing structure, processes, and outcomes.

### Protocol registration

The scoping review protocol was developed in advance and published in PLOS ONE 10.1371/journal.pone.0285916. The protocol followed PRISMA-ScR recommendations [8]. The PRISMA-ScR checklist is provided as an Additional file 1.

### Search strategy

Three databases, PubMed, Cochrane, and Google Scholar, were searched between October and November 2022 for studies reporting on immediate health system response to epidemics in SSA. We used the following search terms: Epidemic OR pandemic AND health system response AND Sub-Saharan Africa.

For the PubMed Literature search, a search strategy was developed using Medical Subject Headings (MeSH terms) and text words related to the population, geographic terms, and the phenomenon of interest/intervention, as shown in Table 1.

**Table 1:** Search terms used PubMed electronic database

Group A: Target population terms (combined by ‘OR’)	Group B: Geographical terms (combined by ‘OR’)	Group C: intervention (combined by ‘OR’)
AND cholera OR diarrhoea OR diarrhoea OR dysentery OR Covid-19 OR leishmaniasis OR kala azar OR trypanosomiasis OR sleeping sickness OR malaria OR polio OR measles OR mumps OR meningitis OR chikungunya OR yellow fever OR dengue OR Ebola OR SARS OR MERS OR influenza OR Zika OR epidemic	AND Angola OR Benin OR Burkina Faso OR Burundi OR Cape Verde OR Central African Republic OR Chad OR Comoros OR Democratic Republic of Congo OR Djibouti OR Equatorial Guinea OR Eritrea OR Ethiopia OR Gambia OR Guinea OR Guinea-Bissau OR Lesotho OR Liberia OR Madagascar OR Malawi OR Mali OR Mauritania OR Mozambique OR Niger OR Rwanda OR Sao Tome and Principe OR Senegal OR Sierra Leone OR Somalia OR Sudan OR Tanzania OR Togo OR Uganda OR Zambia OR Cameroon OR Congo OR Côte d'Ivoire OR Ivory Coast OR Ghana OR Kenya OR Nigeria OR Zimbabwe OR Namibia OR Swaziland OR Botswana OR Gabon OR Mauritius OR South Africa OR Seychelles OR Sub-Saharan Africa OR hard-to-reach areas in Sub-Saharan Africa OR ASAL areas in SSA	Health systems impact OR Health systems resilience OR Health systems performance OR Health systems response OR Health system assessment, OR Immediate health system response, OR Short-term health system response

### Inclusion criteria

The inclusion criteria included empirical studies with either qualitative or quantitative data published in English, as well as countries termed as less developed countries by the United Nations. Systematic and scoping reviews were also considered. Full-text English papers from 2010 to 2024 were considered. This period is critical because most major disease outbreaks occurred during this time.

### Exclusion criteria

Studies were excluded if they were non-empirical (such as editorials or commentaries), conducted outside Least Developed Countries (LDCs), published in languages other than English, or published outside The 2010–2024 period. Additionally, articles lacking full texts, duplicates, or those not aligned with the research objectives were also excluded.

### Quality assessment of included literature

Data search of the CABI Direct database was conducted on 26th October 2022, yielding 36 hits. MeSH terms used to search on CABI Direct included *Epidemic OR disease outbreak AND Health system response AND sub-Saharan Africa.* An abstract and superficial content review was carried out on 1 st November to assess article relevance, yielding 8 results. A detailed literature review was conducted on the same day, and one article was selected from the database.

Data search of the Google Scholar database was conducted on 1 st November 2022, yielding 19,200 hits. The search terms on Google Scholar were “*Health system response to epidemics AND Sub-Saharan Africa* the first ten pages were reviewed. An abstract and superficial content review of the ten pages yielded 12 papers. A detailed interrogation of the 12 papers conducted on 4th November 2022 reduced the number of viable papers for inclusion to 5.

All identified records were exported to a reference management system, Zotero (version 7), where duplicates were removed. Titles and abstracts were screened independently by two reviewers. Potentially relevant full texts were retrieved and assessed against the eligibility criteria. Discrepancies were resolved through discussion until consensus was achieved.

### Evidence extraction and analysis

**Fig. 1:**
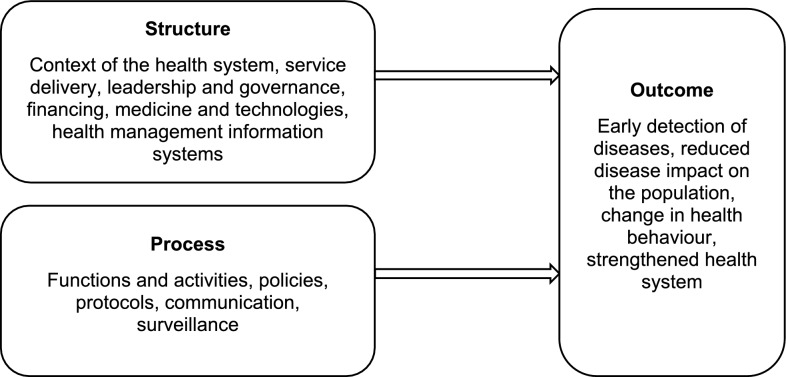
Modified Donabedian framework for the scoping review (source: author’s own)

The review team created a structured data charting form on Excel grounded in the research questions. The conceptual framework guiding this review is the modified Donabedian Framework [9], illustrated in Fig. 1. The framework is commonly used to assess the quality of health services. For this review, a modified framework was used to help understand the different approaches used in immediate health system responses to disease outbreaks. For the review, *Structures* describe the context of the health system and include the WHO building blocks. *The process* will include functions and activities. *Outcomes* will include early detection of disease outbreaks and their impact.

Data extraction was performed independently by one reviewer and verified by two additional reviewers to ensure thoroughness and accuracy. Any disagreements or uncertainties were addressed through discussion and consensus. The charting process was iterative, enabling the inclusion of emerging themes or variables as new insights arose during the review.

All extracted data were compiled into a single evidence matrix, which constituted the foundation for the thematic synthesis presented in the Results section.

Data were analysed thematically using Microsoft Excel (Microsoft 365), using both deductive (framework-based) and inductive methods to identify emerging themes. Initially, findings were categorised according to Donabedian’s three dimensions: structure, process, and outcomes. Each category was then mapped onto the WHO health system building blocks. Finally, results across countries were compared to examine similarities and differences in epidemic response patterns.

Findings were synthesised narratively, supplemented by tables and figures that show trends, the distribution of studies, and cross-country differences. The focus was on identifying actionable insights for strengthening epidemic preparedness and health system resilience in SSA.

## Results

**Fig. 2:**
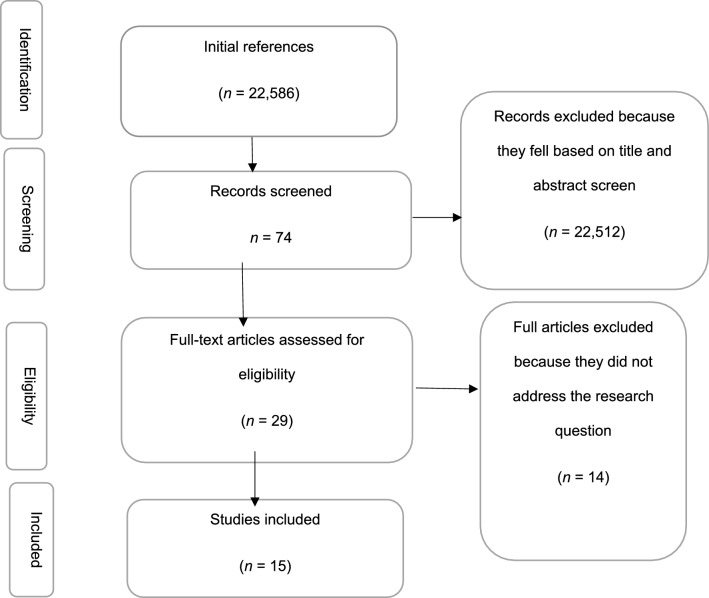
PRISMA flow diagram

A total of 15 studies met the inclusion criteria, as shown in the Preferred Reporting Items for Systematic Reviews and meta-analysis (PRISMA) diagram (Fig. 2), and were included in the final analysis. These studies were conducted between 2014 and 2022 across various sub-Saharan African (SSA) countries, focusing on responses to COVID-19, Ebola Virus Disease (EVD), and other epidemic-prone infections. Most studies were published between 2019 and 2022, reflecting heightened attention to epidemic preparedness during and after the COVID-19 pandemic.

Table 2 summarises the geographical coverage, disease focus, and study designs of the studies included in this scoping review.

**Table 2:** Geographical, methodological, and disease focus of included studies

Study Focus	Countries/Region covered	Disease focus	Study design	Number of studies
Single-country	Rwanda	COVID-19	Descriptive	1
Single-country	Sierra Leone	Ebola Virus Disease (EVD)	Qualitative/Descriptive	2
Single-country	Liberia	Ebola Virus Disease (EVD)	Descriptive/Discussion	2
Single-country	Ethiopia	COVID-19	Mixed methods	2
Single-country	South Africa	COVID-19	Descriptive	1
Single-country	Uganda	COVID-19/Ebola	Mixed methods/Qualitative	2
Single-country	Nigeria	COVID-19	Descriptive/Opinion	3
Single-country	Zambia	COVID-19	Descriptive	1
Regional	Sub-Saharan Africa	Multiple epidemics	Conceptual	1
Regional	West Africa and Democratic Republic of Congo	Ebola Virus Disease	Descriptive	1
Multi-country	Morocco, Uganda, Rwanda, Senegal, Nigeria, Togo	COVID-19	Descriptive	1
Multi-country	Nigeria, Rwanda, South Africa	COVID-19	Descriptive	1
Multi-country (EVD-affected)	Guinea, Liberia, Sierra Leone	Ebola Virus Disease	Qualitative	1
Cross-border	Liberia and Sierra Leone	Ebola Virus Disease	Discussion	1

### Findings

Findings were analysed according to the Donabedian framework, focusing on *structure, processes,* and *outcomes.* Structural factors correspond to the WHO health system building blocks, while process and outcome findings relate to implementation and system effects.

### Structural factors influencing epidemic response

#### Health workforce

Evidence from the reviewed articles highlights the significant impact of epidemics on the health workforce, particularly regarding infection risk, workforce capacity, and system responsiveness. Health workers were disproportionately affected during outbreaks such as COVID-19 and Ebola, with high infection and mortality rates [10]. linked to exposure to patients, including those with undetected or subclinical infections [11]. This elevated risk contributed to widespread fear among healthcare workers, particularly concerning transmission to family members, which in turn affected the effectiveness of the health system response [12].

The literature consistently demonstrates that epidemics exacerbate pre-existing workforce shortages. Increased disease burden, combined with infections among health personnel and inadequate access to personal protective equipment, further constrained already limited human resources [9, 11, 13, 14]. These challenges reduced critical response capacities, including testing and contact tracing. In addition, there was limited prioritisation of the psychological well-being of healthcare workers, with mental health needs largely overlooked despite heightened stress and risk exposure [15].

Adaptation strategies within health systems mainly focused on rapid capacity building and workforce reorganisation. Training of health workers on disease transmission, prevention, and case management was widely implemented at the start of outbreaks [11, 12, 16, 17]. However, the repurposing and redistribution of staff to respond to epidemics often disrupted essential health services and increased workload pressures. Measures such as staff rotation, remote working arrangements, and targeted training were introduced in some settings, reflecting efforts to balance epidemic response with the continuity of care, albeit with varying levels of effectiveness [18].

#### Health financing

Two papers focused on health financing [10, 19].

The reviewed literature indicates that epidemics significantly disrupt health financing systems, often exposing underlying weaknesses in financial risk protection and resource allocation. In response to COVID-19, some countries increased health sector funding, as observed in Ethiopia where budget allocation rose to approximately 10% [9]. More broadly, several countries in sub-Saharan Africa, including Ghana, Kenya, Tanzania, Nigeria, Ethiopia, and Rwanda, implemented or strengthened national health insurance schemes to address gaps in financial protection during health crises [19].

Despite these efforts, epidemics placed substantial strain on financial systems across the region. Increased opportunity costs associated with emergency response measures constrained routine health financing, leading to reduced budgetary allocation for essential health services, shortages of critical medical supplies, and limited resources for procuring essential medicines [19]. Collectively, the evidence suggests that while adaptive financing mechanisms were introduced, they were often insufficient to fully mitigate the systemic pressures imposed by large-scale disease outbreaks.

### Service delivery

The literature consistently shows that epidemics like COVID-19 and Ebola greatly disrupted the delivery and utilisation of health services, exposing critical limitations in health system capacity [10, 13, 15], such as ventilators and strained infrastructure, including limited hospital beds and inadequate isolation facilities, which negatively affected health systems' ability to respond effectively and maintain quality service delivery [10, 19].

Across multiple settings, there was a significant decline in the utilisation of essential health services during the early phases of the outbreaks, including outpatient care, maternal health services, routine immunisation, emergency services, and inpatient admissions [10, 13, 16]. This decline was attributed to both reduced demand for healthcare due to fear of infection and systemic weaknesses in the health system’s resilience [10]. Furthermore, the diversion of resources towards epidemic response often compromised routine service provision, leading to reduced access to care and increased mortality from other conditions such as HIV and malaria [16].

At the same time, health systems adopted adaptive measures to increase service capacity in response to the crises. These included establishing new testing laboratories, expanding healthcare infrastructure through additional facilities, and boosting diagnostic capacity [12, 15, 17, 20]. For example, Ethiopia and other African countries quickly set up laboratory systems, including arbovirus reference laboratories, to support COVID-19 testing during the early stages of the pandemic [17, 20]. Additionally, context-specific treatment protocols, infection prevention and control guidelines, and facility readiness assessment tools were developed to enhance response efforts [17].

### Leadership and governance

The literature review highlights the crucial role of leadership and governance in influencing epidemic preparedness and response in various settings [9, 10, 13, 14, 17, 20–23]. Weak national leadership often correlates with poor coordination, disjointed responses, inconsistent communication, and delays in deploying control measures [16, 21]. These problems are sometimes worsened by the politicisation of decision-making and the challenges of coordinating numerous international actors, which can obstruct rapid information exchange and effective outbreak control [16].

Conversely, strong governance structures enabled more coordinated and effective responses. Many countries established high-level national coordination mechanisms, including presidential advisory committees and task forces, to oversee epidemic response, mobilise resources, and enhance intersectoral collaboration [9, 10, 14, 17, 21, 23]. At the regional level, the creation of the African Task Force for Coronavirus by the Africa Centres for Disease Control and Prevention further supported coordinated action among countries [20]. Evidence also underscores the importance of early preparedness measures, as demonstrated in Guinea, where national task forces and operational centres were set up before cross-border Ebola transmission [21].

Multisectoral and hierarchical coordination structures were commonly adopted to enhance response effectiveness. For example, Ethiopia implemented a three-tiered coordination system, chaired at the highest political level, to guide preparedness and response efforts, including risk assessment, planning, and collaboration with international partners such as WHO18. Additionally, governance responses extended to economic and financing measures, with countries like Nigeria introducing fiscal policies to strengthen health system funding during the pandemic [19]. Overall, the evidence indicates that strong political commitment, coordinated leadership, and effective governance mechanisms are critical in mitigating the spread and impact of epidemics [9].

### Health information systems

The literature emphasises the vital role of health information systems in supporting epidemic preparedness and response, while also revealing ongoing weaknesses across sub-Saharan Africa [16, 17, 19]. Weak health management information systems (HMIS) were linked to issues with data accuracy, timeliness, and reliability, as evidenced by discrepancies in reported COVID-19 cases [19]. These shortcomings weakened evidence-based decision-making during health emergencies.

In addition, inadequate digital infrastructure, including poor telecommunications, limited information technology equipment, and insufficient technical capacity, constrained the ability to track resources effectively, such as stockpiles and service availability [16]. These systemic gaps hindered coordination and real-time response efforts during outbreaks.

In response to these challenges, efforts to strengthen information systems have been observed, particularly following major epidemics. Post-Ebola investments led to the development of partnerships to improve digital health infrastructure and enhance emergency preparedness [16]. At the national level, countries such as Ethiopia established formal information and coordination mechanisms, including a COVID-19 readiness and response Incident Management System, to support data-driven decision-making and response coordination [17].

### Medical products, vaccines, and technologies

The literature shows that epidemics such as COVID-19 have revealed major weaknesses in the availability and management of essential medical products and technologies across sub-Saharan Africa [9, 10, 19]. Widespread shortages of vital supplies, including personal protective equipment, ventilators, medical devices, and testing kits, limited health systems' ability to respond effectively to the pandemic [9, 10, 19]. Additionally, insufficient laboratory infrastructure and limited testing capacity led to delays in diagnosing and managing suspected cases, thereby hampering timely outbreak control efforts [9, 19].

To address these challenges, some countries implemented adaptive logistical and planning mechanisms during outbreaks. For instance, during the Ebola response, Uganda established sub-committees to conduct logistics assessments, identify high-risk districts, and support forecasting and quantification of essential supplies, including medicines, PPE, and laboratory needs [13]. These approaches highlight efforts to strengthen supply chain management and improve preparedness amid resource constraints.

### Processes shaping health system response

The literature highlights various processes and approaches used to manage epidemics across sub-Saharan Africa. Instead of full lockdowns, several countries adopted targeted public health measures, including restrictions on gatherings and movement, school closures, mandatory screening, quarantine, contact tracing, travel restrictions, and improved hygiene practices [6, 9, 12, 15, 16]. Surveillance systems were enhanced through community-based methods, digital platforms, and better border monitoring, such as outbreak response management systems and reporting mechanisms for international arrivals [9, 10, 12, 13, 23].

Community engagement emerged as a critical component of epidemic response, particularly in Ebola-affected settings. Countries leveraged community-based surveillance, social mobilisation, and decentralised contact tracing supported by digital tools such as geospatial mapping, electronic notification systems, and home-based monitoring platforms [12, 13, 16, 17, 21, 22]. Broader coordination mechanisms were also established, including continental strategies for mass screening and national task forces to guide response efforts and research initiatives [9, 14, 22]. In addition, some countries implemented mental health interventions, such as the WHO mhGAP programme, to support frontline workers during outbreaks [11].

Financial and technological adaptations further supported response efforts. Increased health sector funding, improved domestic resource mobilisation, and innovative financing mechanisms were observed in some contexts [9, 19]. Technology played a pivotal role in enhancing response capacity, including the use of telemedicine, social media, digital health platforms, and GPS-enabled systems to support surveillance, communication, and service delivery [11, 14, 16, 18, 21, 22].

### Outcomes of health system response

The literature shows that key public health measures, including contact tracing, thermal screening, quarantine, and case management, were effective in controlling epidemics, even in fragile health systems [9]. Community engagement was also crucial in reducing transmission, especially during the Ebola outbreak, although concerns remain about communities' capacity to sustain effective responses [10, 12].

Digital innovations further strengthened epidemic response, with countries such as Nigeria, Ethiopia, South Africa, and Ghana deploying data-driven platforms, mobile applications, and call centres to enhance surveillance, communication, and timely decision-making, thereby reducing transmission and mortality [19]. However, some interventions had mixed outcomes; for instance, while lockdowns supported disease containment, they also disrupted livelihoods and, in some cases, led to increased transmission following relaxation of restrictions [10, 15].

Post-epidemic and adaptive strategies also produced positive results. Community healing dialogues in Sierra Leone and Liberia reduced stigma and enhanced psychosocial well-being [11], while workflow adjustments during COVID-19 improved aspects of service delivery [18]. Additionally, investments in health infrastructure, including specialised facilities often developed through public–private collaboration, increased health system capacity [19].

## Discussion

The scoping review analysed evidence on short-term health system reactions to epidemics across SSA. It highlighted systemic weaknesses but also showcased examples of agility, innovation, and leadership that contributed to effective containment. Using the Donabedian framework, the findings indicate that although outbreak epidemiological profiles differ, the key determinants of successful responses are mainly structural, centred on governance, funding, workforce, and information systems.

### Overview of key findings

Among the fifteen studies examined, recurring patterns consisted of restricted financing flexibility, shortages of qualified health workers, inadequate intersectoral coordination, and vulnerable data systems. Countries with proactive governance and strong community engagement, earlier such as Rwanda, Uganda, and Ethiopia, demonstrated greater responsiveness and managed the containment of outbreaks.

### Leadership and governance

Lack of leadership at the national level was identified as one of the impediments to containing both the Ebola and COVID-19 outbreaks, leading to poor coordination, inconsistent communication, and a lack of prompt response [16, 23]. This happened despite many countries establishing presidential advisory committees and providing resources to contain the various outbreaks [10, 11, 15, 17, 22, 23]. Due to the novelty of the coronavirus, the African CDC set up an African task force to help countries manage and respond to the outbreak.

Whereas poor leadership can lead to poor outcomes, good leadership can have the opposite effect. Leadership by policymakers and sectoral leaders was noted as being vital in reducing the spread of COVID-19 [10]. During the Ebola outbreak in West Africa, the governments of Liberia, Guinea and Sierra Leone were slow in initiating early emergency response. This was caused by the politicisation of priorities and the increasingly complex network of international actors that made it difficult to share much-needed information [16]. When Ethiopia identified its first cases, the prime minister established a three-layered coordination structure comprising both the public and private sectors. In addition, an incident management team was formed in collaboration with WHO to coordinate risk assessment and guide the development of COVID-specific preparedness and response plans [17]. Countries such as Nigeria implemented fiscal policies that improved funding for the health system [10].

### Health workforce and psychosocial resilience

A recurrent theme across studies was the dual burden faced by health workers, such as physical risk and psychological strain [10–12, 15, 16]. Epidemics not only expose workforce shortages but also erode morale and motivation. While most countries focused on PPE and infection control, few addressed mental health and psychosocial support.

For countries that focused on providing PPE and infection control, their health workers had a lower risk of infection, but this did not prevent the emotional exhaustion caused by extended working hours due to staff shortages and anxiety linked to fear and stigma of infecting family members [10, 12, 15]. Neglecting the psychosocial well-being of the health workers sent an implicit signal that the health workers were viewed mainly as a technical input rather than as individuals facing trauma and risk. Ali et al. [24] noted that there is a relationship between healthcare workers' mental health and the possibility of increased medical errors. A healthcare worker who suffers from anxiety and depression cannot give their full input, remains tired and exhibits an inefficient thought process*.* The HEPR Framework and the Global Strategy on Human Resources for Health underscore workforce resilience as a critical determinant of response capacity [25, 26]. Integrating mental health support, clear safety protocols, surge workforce planning, and training into national preparedness strategies can enhance both individual and system resilience [13, 15, 17, 18].

Investment in community health workers (CHWs) proved crucial as well. Their engagement in surveillance, risk communication, and contact tracing bridged the gap between formal health systems and communities, particularly in rural and hard-to-reach areas. Institutionalising CHWs within epidemic response frameworks offers a scalable model for rapid mobilisation.

### Financing and resource agility

Many SSA countries experienced severe financial bottlenecks during the epidemic onset [10, 19]. Health budgets were insufficiently flexible to allow rapid reallocation or procurement, and reliance on donor funding slowed response time. Only a few, such as Ethiopia and Ghana, implemented adaptive financing measures or leveraged national insurance mechanisms [19].

The HEPR Framework and the World Bank’s Pandemic Fund both stress the importance of predictable, flexible, and sustained financing. Creating dedicated contingency funds, supported by national legislation, could enhance financial readiness. Fiscal decentralisation, granting subnational units authority to reallocate funds during emergencies, was a distinguishing feature of more agile systems, such as Rwanda and Nigeria [10].

### Service delivery and continuity of care

The review revealed that during epidemics, routine service delivery frequently declined because of workforce diversion and resource shortages. Shortage of essential supplies such as ventilators, beds, and isolation centres determined the quality of services provided and how the health facilities responded to the different epidemics [10, 19]. Services for other conditions other than COVID-19 and Ebola were almost non-existent, which meant routine services such as vaccination, emergency services, inpatient admissions, and maternal health services declined, partially leading to higher mortality from other diseases such as HIV and malaria [10, 16]. This was also driven by decreased demand for healthcare services and a lack of resilience in the healthcare system.

The results show that expansion of health services to adequately manage outbreaks, such as the construction and expansion of testing laboratories and additional hospitals, occurred during the Ebola and COVID-19 outbreaks. This allowed for rapid testing and contact tracing of potentially infected people and provided more room for treatment and management of the different diseases [12, 15, 17, 20]. Countries such as Ethiopia developed treatment and infection-prevention protocols to ensure uniformity in care and facility-readiness checklists during the COVID-19 outbreak [17, 20].

### Information systems and digital innovation

Health information systems play a vital role during epidemics. The results from the review highlighted gaps, especially during the COVID-19 outbreak, where there were discrepancies regarding the actual number of cases reported [19]. Information on stockpiles and available resources for refilling them was also hindered due to lack of information technology equipment and expertise to use what was available [16]. Partnerships in relation to data collection and management were developed in West Africa during the Ebola outbreak to strengthen digital health infrastructure and emergency preparedness, and the development of a COVID-19 readiness and response incident management system at the national level in Ethiopia [16, 17].

Beyond national systems, regional coordination was pivotal. The Africa CDC and the African Union played instrumental roles in harmonising surveillance protocols, facilitating resource mobilisation, and promoting collective learning. Countries that aligned their national strategies with regional frameworks benefited from faster access to technical assistance and shared data systems. However, duplication and limited interoperability between global and regional mechanisms remain challenges. Strengthening regional coordination requires clearer alignment between regional bodies and national public health institutes, along with investment in shared research platforms and laboratory networks.

### Policy implications

From a policy perspective, the review highlights two priority actions:

1. Formalise preparedness as a core component of health policy and law.
2. Epidemic response should move from reactive emergency measures to established policies integrated into national health legislation and disaster frameworks. This approach guarantees consistent leadership and accountability through political changes.
3. Invest in governance and data systems as essential capabilities. Enhancing epidemic preparedness involves upgrading decision-making frameworks and information infrastructure. Digital integration and transparent data systems are crucial to ensuring prompt responses.

### Contribution to policy and practice

This review enhances the discussion on health system resilience by showing that immediate responses can lead to sustained reforms. It connects outbreak management with policy institutionalisation, providing both a conceptual and an empirical basis for translating emergency experiences into broader health system improvements. The successes identified in this study, such as multisectoral coordination, adaptive workforce strategies, and early containment, reflect broader patterns observed in other regions of the world. Cross-regional analyses have shown that whole-of-government responses and community engagement were essential in maintaining service continuity across Africa, Asia, and Latin America during COVID-19 [27].

The findings directly support the WHO HEPR Framework and the Africa CDC Regional Health Security Agenda, offering regionally tailored insights on governance, financing, and digital transformation. They highlight that resilience is an ongoing policy process rather than a fixed trait, necessitating adaptive leadership, investments in human and information systems, and the fostering of trust between governments and communities.

## Limitations

While we strived to capture all relevant literature, a potential limitation to our review is the use of the term “short-term health system responses.” We may have missed relevant articles that describe the concept using different terms.

## Conclusions

The results of this scoping review identified strong evidence for increased integration at different levels of the health system to advance early response to epidemics. Several high-priority elements have been identified that worked in various countries, enabling them to have more robust health system responses at the national and local levels during emergent crises. This includes pandemic preventive measures, rather than a full lockdown of their economies, and the use of different forms of technology; all of this, supplemented by the health system, helped contain the spread of disease.

The scoping review highlights a significant gap in the literature regarding the integration of health systems and leadership effectiveness during epidemic responses. While there is strong evidence supporting increased integration at various levels of the health system to enhance early responses to epidemics, existing studies primarily focus on specific elements that have proven effective in certain countries. However, there is a lack of comprehensive analysis on how these elements can be universally applied or adapted across different contexts, particularly in low-resource settings.

Moreover, although the review identifies critical factors such as technology use and community mobilisation as essential for improving health outcomes during epidemics, it does not sufficiently explore the systemic barriers that hinder the implementation of these strategies. For instance, while poor leadership contributes to delayed responses, there is limited examination of how leadership training and capacity-building initiatives could mitigate these challenges. Additionally, the review emphasizes the need for better coordination between national and sub-national levels but fails to provide insights into specific frameworks or models that could facilitate this integration.

Overall, while the themes and capacities identified in the literature provide a foundational understanding for refining short-term health system responses to epidemics, a critical need remains for more robust research to address these gaps. Future studies should focus on developing actionable frameworks for integrating technology and community participation within health systems while also enhancing leadership capacity to improve preparedness and response to epidemics.

## Supplementary Information

Additional file1

## Data Availability

All data used in this review are publicly available in the cited literature and included in this manuscript.

## Abbreviations

CHWs: Community Health Workers
Covid-19: Coronavirus
EVD: Ebola Virus Disease
HMIS: Health Management Information Systems
LDCs: Least developed countries
LMICs: Low- and middle-income countries
MeSH terms: Medical Subject Headings
PRISMA: Preferred Reporting Items for Systematic Reviews and Meta-Analysis
SARS: Severe Acute Respiratory Syndrome
SSA: Sub-Saharan Africa
WHO: World Health Organisation

## References

[1] Naveed N, Ahmad K, Majeed H, Qureshi K, Ahmad I, Awan MF, et al. The global impact of COVID-19: a comprehensive analysis of its effects on various aspects of life. Toxicol Res. 2024;13:tfae045. 10.1093/toxres/tfae045. 10.1093/toxres/tfae045 PMC10964844 38545435

[2] IFRC. Out of reach: remote and hard‑to‑access populations. 2018. https://www.ifrc.org/sites/default/files/2021-09/C-03-WDR-2018-3-reach.pdf

[3] Garg R, Bhargava A, Singh SK. Capacity building in public health emergency management: a crucial pillar for global health security. NMO J. 2024;18:28. 10.4103/JNMO.JNMO_10_24.

[4] Paintsil E. COVID-19 threatens health systems in Sub-Saharan Africa: the eye of the crocodile. J Clin Invest. 2020;130:2741–4. 10.1172/JCI138493. 32224550 10.1172/JCI138493 PMC7260015

[5] Cohen ML. Changing patterns of infectious disease. Nature. 2000;406:762–7. 10.1038/35021206. 10963605 10.1038/35021206

[6] Cable J, Heymann DL, Uzicanin A, Tomori O, Marinissen MJ, Katz R, et al. Pandemic diseases preparedness and response in the age of COVID-19—a symposium report. Ann N Y Acad Sci. 2021;1489:17–29. 10.1111/nyas.14534. 33155324 10.1111/nyas.14534

[7] Ali M, Lopez AL, Ae You Y, Eun Kim Y, Sah B, Maskery B, et al. The global burden of cholera. Bull World Health Org. 2012;90:209–18. 10.2471/BLT.11.093427. 22461716 10.2471/BLT.11.093427 PMC3314202

[8] The PRISMA 2020 statement: an updated guideline for reporting systematic reviews | The BMJ. https://www.bmj.com/content/372/bmj.n71.short. Accessed 5 June 2025

[9] Voyce J, Gouveia M, Medinas M, Santos A, Ferreira R. A Donabedian model of the quality of nursing care from nurses’ perspectives in a Portuguese hospital: a pilot study. J Nurs Meas. 2015;23:474–84. 10.1891/1061-3749.23.3.474. 26673771 10.1891/1061-3749.23.3.474

[10] Abagero A, Ragazzoni L, Hubloue I, Barone-Adesi F, Lamine H, Addissie A, et al. A review of COVID-19 response challenges in Ethiopia. Int J Environ Res Public Health. 2022;19:11070. 10.3390/ijerph191711070. 36078785 10.3390/ijerph191711070 PMC9518440

[11] Ajisegiri W, Odusanya O, Joshi R. COVID-19 outbreak situation in Nigeria and the need for effective engagement of community health workers for epidemic response. Glob Biosecur. 2020. 10.31646/gbio.69.

[12] Abramowitz SA, McLean KE, McKune SL, Bardosh KL, Fallah M, Monger J, et al. Community-centered responses to Ebola in urban Liberia: the view from below. PLoS Negl Trop Dis. 2015;9:e0003706. 10.1371/journal.pntd.0003706. 25856072 10.1371/journal.pntd.0003706 PMC4391876

[13] Aceng J, Ario A, Muruta A, Makumbi I, Nanyunja M, Komakech I, et al. Uganda’s experience in Ebola virus disease outbreak preparedness, 2018–2019. Glob Health. 2020. 10.1186/s12992-020-00548-5. 10.1186/s12992-020-00548-5 PMC7081536 32192540

[14] Dean L, Cooper J, Wurie H, Kollie K, Raven J, Tolhurst R, et al. Psychological resilience, fragility and the health workforce: lessons on pandemic preparedness from Liberia and Sierra Leone. BMJ Glob Health. 2020;5:e002873. 10.1136/bmjgh-2020-002873. 32988928 10.1136/bmjgh-2020-002873 PMC7523196

[15] Amzat J, Aminu K, Kolo VI, Akinyele AA, Ogundairo JA, Danjibo MC. Coronavirus outbreak in Nigeria: burden and socio-medical response during the first 100 days. Int J Infect Dis. 2020;98:218–24. 10.1016/j.ijid.2020.06.067. 32585282 10.1016/j.ijid.2020.06.067 PMC7307993

[16] Lal A, Ashworth HC, Dada S, Hoemeke L, Tambo E. Optimizing pandemic preparedness and response through health information systems: lessons learned from Ebola to COVID-19. Disaster Med Public Health Prep. 2022;16:333–40. 10.1017/dmp.2020.361. 33004102 10.1017/dmp.2020.361 PMC7642496

[17] Lanyero B, Edea ZA, Musa EO, Watare SH, Mandalia ML, Livinus MC, et al. Readiness and early response to COVID-19: achievements, challenges and lessons learnt in Ethiopia. BMJ Glob Health. 2021;6:e005581. 10.1136/bmjgh-2021-005581. 34112648 10.1136/bmjgh-2021-005581 PMC8193696

[18] Lombe DC, Mwaba CK, Msadabwe SC, Banda L, Mwale M, Pupwe G, et al. Zambia’s National Cancer Centre response to the COVID-19 pandemic—an opportunity for improved care. ecancermedicalscience. 2020;14:1051. 10.3332/ecancer.2020.1051. 32565904 10.3332/ecancer.2020.1051 PMC7289614

[19] Amu H, Dowou RK, Saah FI, Efunwole JA, Bain LE, Tarkang EE. COVID-19 and health systems functioning in Sub-Saharan Africa using the “WHO building blocks”: the challenges and responses. Front Public Health. 2022. 10.3389/fpubh.2022.856397. 35444973 10.3389/fpubh.2022.856397 PMC9013894

[20] Lucero-Prisno DE, Adebisi YA, Lin X. Current efforts and challenges facing responses to 2019-nCoV in Africa. Glob Health Res Policy. 2020;5:21. 10.1186/s41256-020-00148-1. 32391440 10.1186/s41256-020-00148-1 PMC7200322

[21] Bedson J, Jalloh MF, Pedi D, Bah S, Owen K, Oniba A, et al. Community engagement in outbreak response: lessons from the 2014–2016 Ebola outbreak in Sierra Leone. BMJ Glob Health. 2020;5:e002145. 10.1136/bmjgh-2019-002145. 32830128 10.1136/bmjgh-2019-002145 PMC7445350

[22] Nachega JB, Atteh R, Ihekweazu C, Sam-Agudu NA, Adejumo P, Nsanzimana S, et al. Contact tracing and the COVID-19 response in Africa: best practices, key challenges, and lessons learned from Nigeria, Rwanda, South Africa, and Uganda. Am J Trop Med Hyg. 2021;104:1179–87. 10.4269/ajtmh.21-0033. 33571138 10.4269/ajtmh.21-0033 PMC8045625

[23] Ross E. Command and control of Sierra Leone’s Ebola outbreak response: evolution of the response architecture. Philos Trans R Soc Lond B Biol Sci. 2021;372:20160306. 10.1098/rstb.2016.0306. 10.1098/rstb.2016.0306 PMC5394644 28396477

[24] Ali SM, Nausheen S. Psychosocial impact of COVID-19 on healthcare workers. Sultan Qaboos Univ Med J. 2022;22:82–90. 10.18295/squmj.4.2021.067. 35299803 10.18295/squmj.4.2021.067 PMC8904111

[25] WHO. Strengthening health emergency prevention, preparedness, response and resilience. 2023. https://cdn.who.int/media/docs/default-source/emergency-preparedness/who_hepr_wha2023-21051248b.pdf

[26] WHO. Global strategy on human resources for health: workforce 2030. 2016. chrome-extension://efaidnbmnnnibpcajpcglclefindmkaj/https://iris.who.int/server/api/core/bitstreams/3ef6ee65-42fa-4d2b-9c75-d55b2df17f9a/content

[27] Guha M, Tanugi-Carresse AC, Mullen L, Bennett S, Wanyenze RK, Prado AM, et al. Maintaining essential health services during COVID-19: cross-country lessons of health system resilience from Asia, Sub-Saharan Africa and Latin America. BMJ Glob Health. 2023. 10.1136/bmjgh-2023-013392. 10.1136/bmjgh-2023-013392 PMC12826116 41083317

